# Tobacco-Related Alterations in Airway Gene Expression are Rapidly Reversed Within Weeks Following Smoking-Cessation

**DOI:** 10.1038/s41598-019-43295-3

**Published:** 2019-05-06

**Authors:** Kahkeshan Hijazi, Bozena Malyszko, Katrina Steiling, Xiaohui Xiao, Gang Liu, Yuriy O. Alekseyev, Yves-Martine Dumas, Louise Hertsgaard, Joni Jensen, Dorothy Hatsukami, Daniel R. Brooks, George O’Connor, Jennifer Beane, Marc E. Lenburg, Avrum Spira

**Affiliations:** 1 Department of Medicine, Section of Computational Biomedicine, Boston University School of Medicine, Boston, Massachusetts United States; 2 Bioinformatics Program, Boston University, Boston, Massachusetts United States; 3 The Pulmonary Center, Boston University School of Medicine, Boston, Massachusetts United States; 4 Department of Epidemiology, School of Public Health, Boston University, Boston, Massachusetts United States; 5 Department of Pathology and Laboratory Medicine, Boston University School of Medicine, Boston, Massachusetts United States; 6 Tobacco Research Programs, University of Minnesota, Minneapolis, Minnesota United States; 7 Johnson & Johnson Innovation Center, Cambridge, Massachusetts United States

**Keywords:** Microarrays, Risk factors

## Abstract

The physiologic response to tobacco smoke can be measured by gene-expression profiling of the airway epithelium. Temporal resolution of kinetics of gene-expression alterations upon smoking-cessation might delineate distinct biological processes that are activated during recovery from tobacco smoke exposure. Using whole genome gene-expression profiling of individuals initiating a smoking-cessation attempt, we sought to characterize the kinetics of gene-expression alterations in response to short-term smoking-cessation in the nasal epithelium. RNA was extracted from the nasal epithelial of active smokers at baseline and at 4, 8, 16, and 24-weeks after smoking-cessation and put onto Gene ST arrays. Gene-expression levels of 119 genes were associated with smoking-cessation (FDR < 0.05, FC ≥1.7) with a majority of the changes occurring by 8-weeks and a subset changing by 4-weeks. Genes down-regulated by 4- and 8-weeks post-smoking-cessation were involved in xenobiotic metabolism and anti-apoptotic functions respectively. These genes were enriched among genes previously found to be induced in smokers and following short-term *in vitro* exposure of airway epithelial cells to cigarette smoke (FDR < 0.05). Our findings suggest that the nasal epithelium can serve as a minimally-invasive tool to measure the reversible impact of smoking and broadly, may serve to assess the physiological impact of changes in smoking behavior.

## Introduction

Tobacco smoke remains the leading preventable cause of death in the United States, causing more than 440,000 premature deaths each year^1^. Although the risk of tobacco-related death greatly decreases with cessation of use^2^, smokers remain at an elevated risk for many lung diseases in the United States despite quitting. Several studies have shown that smoking creates airway-wide molecular alterations throughout the respiratory tract and that airway epithelial gene expression is dramatically altered in smokers^3^. In addition, cross-sectional studies comparing bronchial epithelial gene expression in current, former and never smokers have shown that most smoking-related gene-expression changes reverse following smoking-cessation, while a subset seem persistently altered in even long-term former smokers^4,5^.

While we have described the degree to which smoking-related gene-expression changes are reversible years to decades after smoking-cessation, the behavior of these genes immediately after smoking-cessation and the time it takes for the genes to revert to normal remain unclear. Given the difficulty of collecting longitudinal samples using fiberoptic bronchoscopy and our previous observation of similar smoking-induced gene-expression changes in the nasal and bronchial epithelium, we sought to leverage the nasal epithelium as a less invasive site for measuring the physiological response to tobacco-smoke exposure^6–8^, thereby allowing for the longitudinal characterization of the temporal dynamics of the smoking induced changes among individuals who have recently quit smoking.

## Results

### Characteristics of the study population

**Figure 1:**
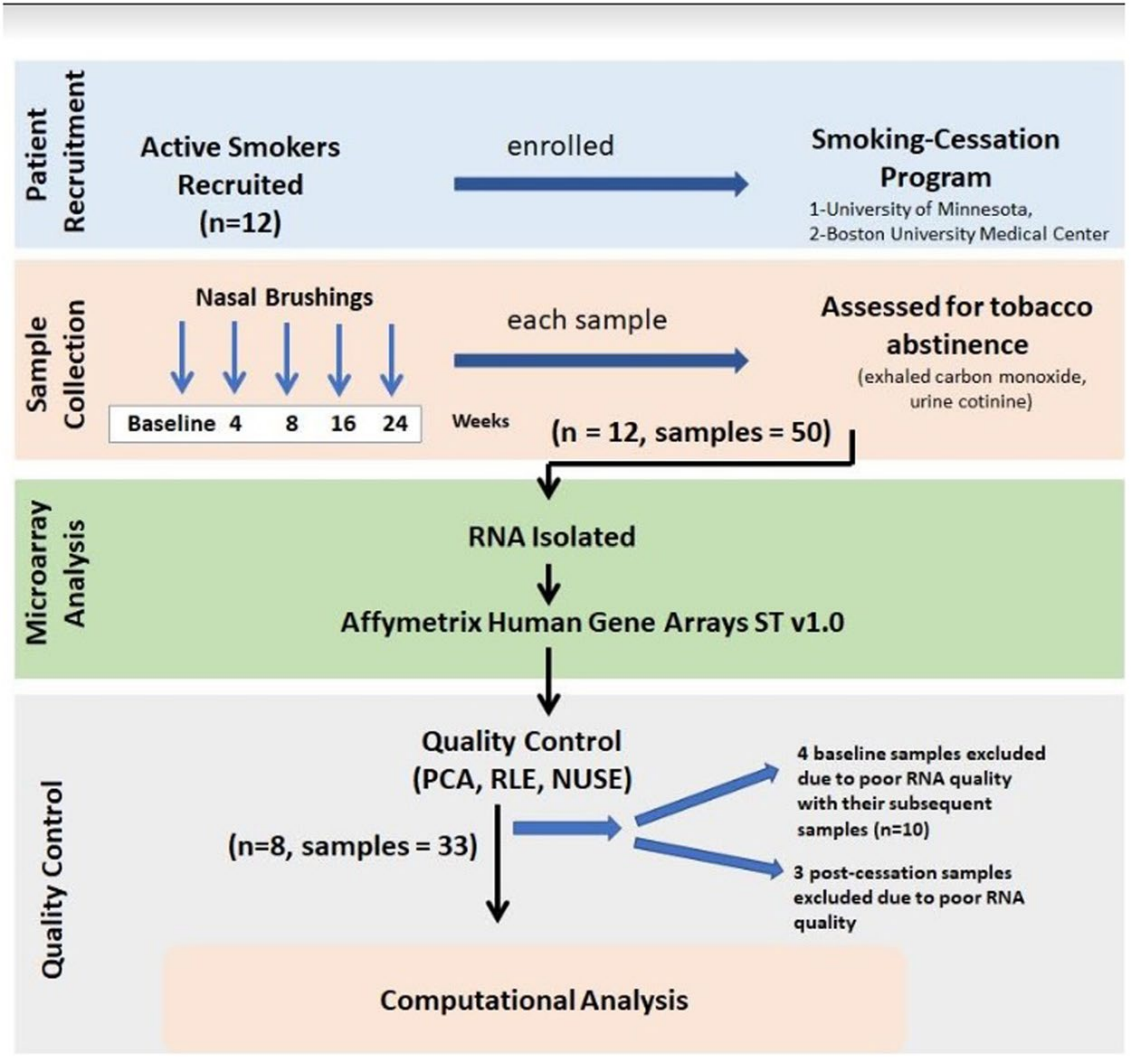
Study design and sample collection. Nasal epithelial brushings were obtained from active smokers enrolled in smoking-cessation programs at Boston University Medical Center (BUMC) and the University of Minnesota (UMN). Samples were obtained at baseline and at 4, 8, 16 and 24 weeks post-smoking-cessation. Initially we obtained only 50 samples from 12 active smokers as some of the participants did not show up for all the post smoking-cessation follow ups (n = 10). Urine cotinine, exhaled carbon monoxide or serum anatabine and anabasine levels were used in the BUMC and UMN cohorts to assess tobacco abstinence. RNA isolated from nasal brushings was processed and hybridized to Affymetrix GeneST arrays. Array quality was assessed using principal component analysis (PCA) resulting in 33 samples from 8 individuals for further analysis.

A total of 12 subjects (50 samples) were initially eligible for the analysis (Fig. 1). Gene-expression data from 17 of the 50 samples were excluded from data analysis. Of these 17 samples, seven were of low quality (See Methods). In order to capture the gene expression differences between pre- and post-cessation, we required each patient to have a baseline sample prior to smoking-cessation. Four of the seven low-quality samples were baseline samples, and therefore, we excluded all samples from these subjects (n = 10). After this sample filtering step, a total of 33 samples from 8 subjects, who had baseline and at least 3 follow-up visits, were included in the analysis (Fig. 1). Demographics (including age, gender, and cigarettes per day pre-smoking-cessation) for these subjects are shown for in Table 1. The range of smoking duration prior to quitting was 2–41 years. Prior to smoking-cessation, smoking intensity was 7–17 cigarettes per day (mean = 13.2; SD = 3.4).

**Table 1:** Characteristics of the 8 individuals (33 samples) who recently quit smoking and from whom gene expression data was extracted to assess gene expression kinetics.

Cohort	Patient	Post-cessation samples available (weeks)	Age (yrs)	Gender	RIN (Mean ± standard deviation)	Cigarettes per day (baseline)
BUMC	107	0, 4, 16, 24	30	Female	7.2 ± 2.0	15
BUMC	116	0, 4, 8	44	Female	6.4 ± 0.9	10
BUMC	206	0, 4, 8, 16, 24	55	Male	6.8 ± 0.9	7.5
UMN	205	0, 16, 24	26	Female	5.9 ± 1.7	12
UMN	208	0, 4, 8, 16, 24	32	Female	5.8 ± 1.8	14
UMN	227	0, 4, 8, 24	64	Male	4.5 ± 1.8	20
UMN	243	0, 4, 8, 16, 24	64	Female	6.5 ± 1.3	20
UMN	265	0, 4, 8, 16	57	Male	5.2 ± 1.6	10

Abbreviation RIN stands for RNA Integrity Number for RNA quality.

### Transcriptomic response of the nasal epithelium to smoking-cessation

**Figure 2:**
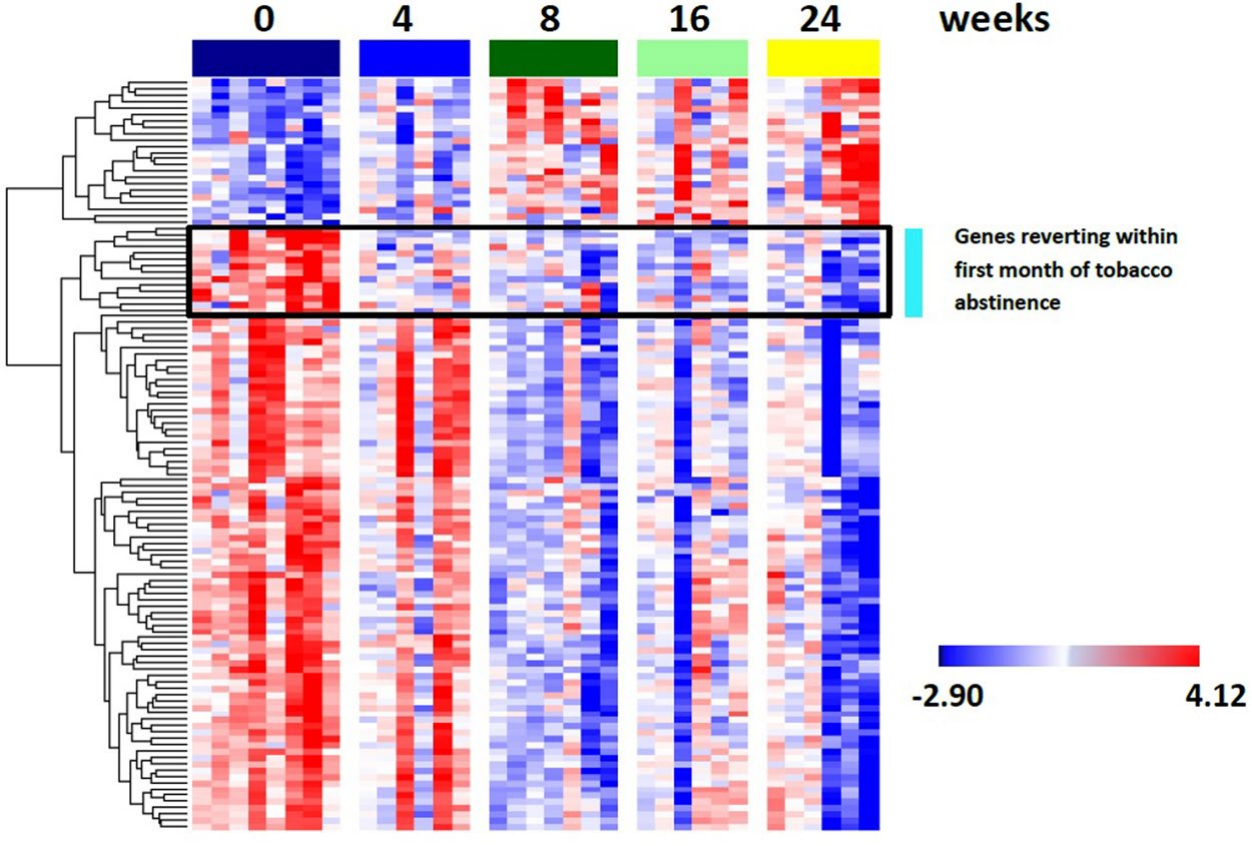
Gene expression dynamics following smoking-cessation. The 119 genes whose expression profiles are associated with the duration of tobacco abstinence (FDR < 0.05 and FC ≥ 1.7) were clustered by hierarchical clustering. This identified three main patterns of smoking-cessation associated gene expression: a cluster of genes induced by the 8-week time point, a cluster of genes repressed by the 4-week time point, and a final cluster of genes repressed by the 8-week time point. Gene Expression levels were adjusted for the effect of RIN and patient effect prior to hierarchical clustering.

To determine the acute effects of smoking-cessation on nasal epithelial gene expression, we modeled gene expression as a function of log-transformed time. After controlling for RNA integrity and a random patient effect, the expression levels of 119 genes were associated with time since smoking-cessation (false discovery rate (FDR) < 0.05 and fold change (FC) ≥ 1.7) (Fig. 2). The expression levels of 23 genes increased over 24 weeks of smoking-cessation, and 96 decreased over the time period. Using hierarchal clustering we identified that the majority of genes changed within the 8-weeks following smoking-cessation while a subset of these genes changed as early as 4-weeks following smoking-cessation, suggesting that some genes are more rapidly altered compared to others following smoking-cessation (see Supplementary Table S1). Quantitative real time polymerase chain reaction (RT-PCR) analysis was used to confirm the differential expression of three genes that reversed with different kinetics following smoking-cessation (CYP1A1, CYP1B1, ALDH3A1) (see Supplementary Fig. S1).

In addition, to assess potential non-linear changes in gene expression in response to short-term smoking-cessation, we first modeled time as a binary factor that transitioned from a classification of “baseline” to a classification of “recovered” at each of the possible timepoints. Additionally, we modeled time as a binary factor with either the four- and/or eight-week timepoints classified as “recovering” with the other timepoints classified as “baseline” to explore transient gene expression differences associated with smoking cessation. Only the model where timepoints before 8-weeks were modeled as “baseline” and the 8-week and later timepoints were modeled as “recovered” (e.g. 00111 model) yielded more than a single differentially expressed gene (see Supplementary Table S2 in the online supplement and Supplementary Data S1). This finding is consistent with the results from the model with time as a continuous variable in which most of the differentially expressed genes changed expression levels between the four- and eight-week timepoints. Methods yield highly overlapping lists of significantly differentially expressed genes (92.8%) (see Supplementary Table S2).

### Enrichment of smoking-cessation genes in independent smoking datasets

**Figure 3:**
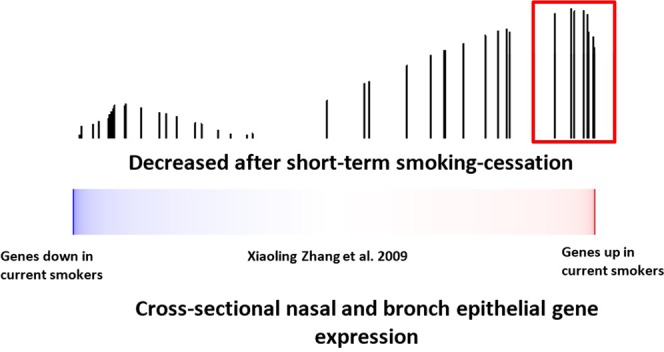
The relationship between nasal epithelial gene expressions associated with smoking-cessation and smoking-induced changes in nasal and bronchial epithelium gene expression. Nasal epithelial genes whose expression decreases upon smoking-cessation in a longitudinal study were concordantly enriched among genes whose expression were higher in current smokers compared to never smokers FDR_GSEA_ < 10^−3^). The color bar indicates ranking of genes with response to induction/repression when comparing current verses never smokers. Vertical bars indicate genes substantially decreased with smoking-cessation in the current study. The height of the bars indicates the running GSEA enrichment score.

**Figure 4:**
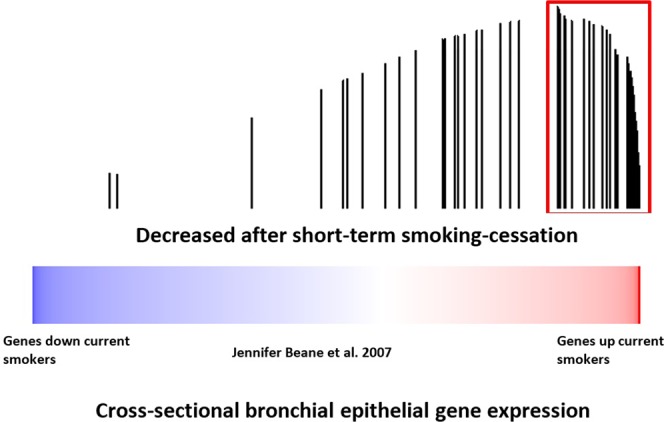
The relationship between nasal epithelial gene expressions associated with smoking-cessation and cessation-induced changes in cross-sectional bronchial epithelium gene expression Nasal epithelial genes whose expression decreased after smoking-cessation were concordantly enriched among bronchial epithelial genes whose expression levels were lower in long-term former smokers compared to current smokers (FDR < 10^−3^). The color bar indicates ranking of genes with response to gene expression changes when comparing current verses long term former smokers. Vertical bars indicate genes substantially decreased with smoking-cessation in the current study. The height of the bars indicates the running GSEA enrichment score.

To determine whether smoking-cessation gene-expression alterations are among the genes that differ between never and current smokers, we examined the expression of the gene we identified as changing with short-term smoking-cessation in an independent dataset of paired bronchial and nasal epithelial samples obtained from current and never smokers (GSE16008)^8^ using gene set enrichment analysis (GSEA)^8,9^. Genes whose expression levels decrease in the nasal epithelium of recent former smokers (47 of 96 genes) were enriched among the genes whose expression levels increase in nasal and bronchial epithelium of current smokers compared to never smokers in this dataset (FDR_GSEA_ < 10^−3^; Fig. 3) with 10 of the 47 genes down regulated with short-term smoking-cessation strongly contributing to the observed enrichment (GSEA leading edge genes). We also connected our dataset to one containing a cohort of former smokers (GSE7895) (Fig. 4). This cross-sectional dataset consists of microarray data derived from of cytologically normal bronchial epithelial brushings obtained from never, current and long-term former smokers (months since quit 145.2 ± 162.82)^5^. Genes whose nasal epithelial expression levels decreased with short-term smoking-cessation were enriched among genes that increase in current smokers compared to never smokers in the bronchial epithelium (FDR_GSEA_ < 10^−3^) (data not shown) and among genes whose expression levels decreased in the bronchial airway with longer-term smoking-cessation (FDR_GSEA_ < 10^−3^) (Fig. 4). Based on GSEA results, a total of 50 of the 96 genes down-regulated with short-term smoking-cessation were enriched among the genes differentially expressed between current and former smokers. Of these, 30 were strongly enriched between both datasets (leading edge genes). These findings suggest that nasal epithelial genes whose expressions levels are altered in response to tobacco exposure and change following short-term smoking-cessation are concordant with independent nasal and bronchial airway datasets.

**Figure 5:**
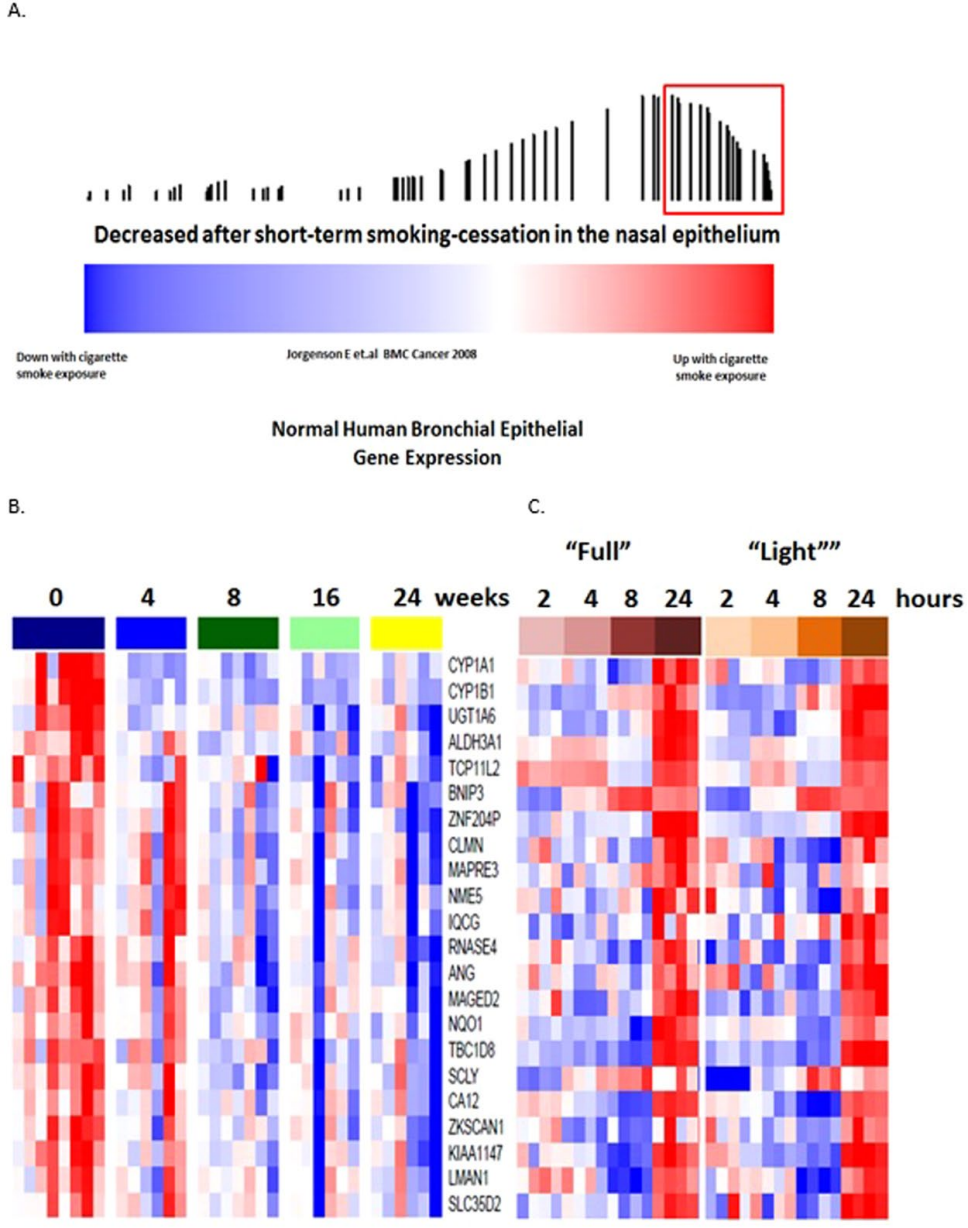
Genes that reverse with short-term smoking-cessation are enriched among genes that are induced with rapid kinetics with acute whole cigarette smoke exposure *in vitro*. Nasal epithelial genes whose expression are reversed with smoking-cessation were concordantly enriched among genes that whose expression are induced of NHBE to CS for 15 minutes invitro (FDR_GSEA_ < 0.02). The color bar (**A**) indicates the association of NHBE gene expression in response to cigarettes smokes exposure (red = increased gene expression after CS exposure; blue = decreased gene expression after CS smoke exposure). Vertical bars indicate genes associated with smoking-cessation. The height of the bars indicates the running GSEA enrichment score. Expression of the common leading edge genes between the two datasets (red block in a) is shown in both panels b and c. Leading edge genes that are rapidly reversible upon short-term smoking-cessation in in the nasal epithelium (**B**) are enriched among the genes that are induced following 15 minute *in vitro* exposure (**C**) of NHBE to smoke from either “Full flavor” or “Light” tobacco cigarettes over 24 hours. This data demonstrates that that gene-expression response to tobacco exposure occurs with rapid kinetics and these genes are rapidly reversible with short-term smoking-cessation.

Given the rapid kinetics of the gene-expression response to smoking-cessation, we sought to determine whether they are also rapidly changed in response to acute cigarette smoke exposure. We examined the expression of the genes altered by smoking-cessation in a previous published study of cultured normal human bronchial epithelial (NHBE) cells treated with whole cigarette smoke for 15 minutes (GSE10700) in which gene expression was assessed at 2, 4, 8 and 24 hours after exposure and found that expression levels which decrease following smoking-cessation are enriched among expression levels that were increased in NHBEs exposed to cigarette smoke exposure FDR_GSEA_ ≤ 0.02 (Fig. 5). Based on GSEA results, a total of 67 of 96 down-regulated genes with short-term smoking-cessation were enriched among the genes up-regulated with 15-min cigarette smoke exposure with 22 leading edge genes showing strong enrichment between the two datasets. This finding suggests that many of the rapidly reversible genes associated with tobacco-abstinence are rapidly inducible with tobacco smoke exposure. These data also suggest that exposure to and subsequent abstinence from tobacco smoke causes select genes to rapidly turn on and off, suggesting that ongoing exposure to tobacco smoke is required for the continuous gene-expression alteration associated with tobacco smoke.

### Functional kinetics of genes altered with tobacco-recovery

**Figure 6:**
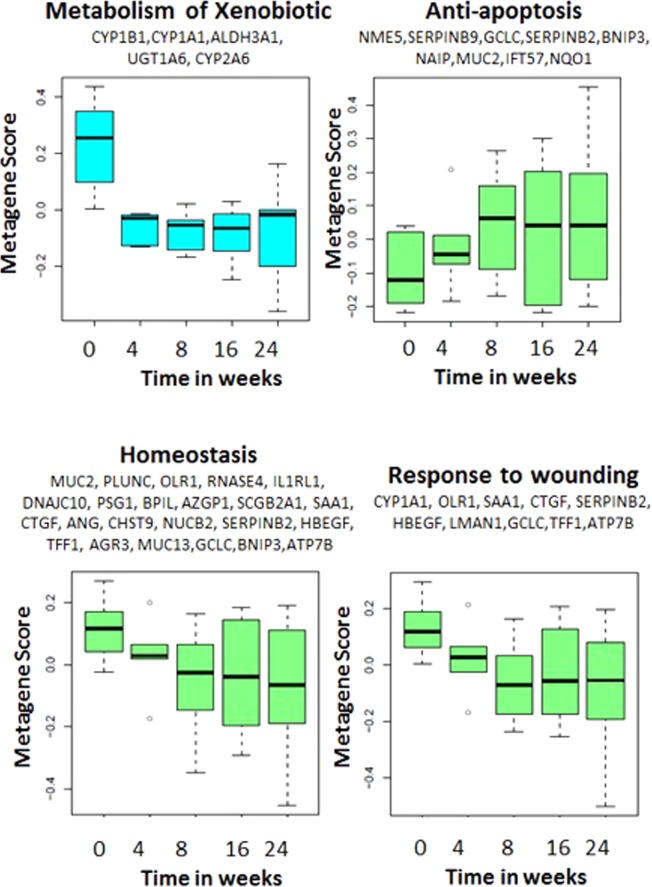
Kinetics of genes altered with smoking-cessation and functional categories. For each of the seven functional categories that are over represented among the 119 genes that change after smoking-cessation, we calculated a metagene score to summarize the behavior of the genes. The metagene score for the expression of genes in four of these functional categories substantially associated with time since smoking-cessation are shown here. Of the functional categories, genes involved in xenobiotic metabolism were altered within four weeks of smoking-cessation, while the other categories changed more gradually.

We identified 7 broad functional categories over represented among the 119 genes associated with smoking-cessation using DAVID (see Supplementary Table S3). These include genes associated with metabolism of xenobiotics, anti-apoptosis, homeostatic processes, response to wounding and purine nucleotide metabolism. To examine the kinetics with which the expression of genes in these functional categories change following smoking-cessation, we calculated a metagene score to summarize the behavior of the genes in each of these functional categories. Using this approach, we identified time-dependent changes in these functional categories (P-value < 0.05) of which only four are shown (Fig. 6). Among these changes we found that genes involved in the metabolism of xenobiotics were down-regulated with tobacco recovery and, unlike the remaining categories reverted within 4 weeks of tobacco abstinence. This category includes genes involved in the metabolism of carcinogenic components of cigarette smoke. Genes implicated in homeostatic processes and response to wounding were more gradually down-regulated with smoking-cessation. Genes involved in inhibition of apoptosis were up-regulated in response to tobacco abstinence within 8 weeks of smoking-cessation.

## Discussion

Tobacco smoke exposure causes changes in airway gene expression with reversal of most of these changes among former smokers. It remains unknown how quickly these genes revert to baseline following smoking-cessation. Differences in gene-expression dynamics following cessation could also identify distinct biological processes that are activated in response to and recovery from tobacco smoke exposure. Using relatively non-invasive nasal airway epithelial cells from 8 active smokers enrolled in smoking-cessation programs over 24 weeks; we have captured the acute changes that occur upon smoking-cessation. The majority of genes we identified changed between 4- and 8-weeks following tobacco abstinence. A small subset of genes (mostly involved in xenobiotic metabolism) changed before 4 weeks. Most genes showed a down-regulation in their expression level in response to smoking-cessation, which is consistent with previous findings that have shown that many genes are up-regulated in response to tobacco smoke exposure.

Our results show that 11.8% of genes associated with smoking-cessation change before 4 weeks and 88.2% of genes change between 8 weeks and that these genes corresponded to distinct biological processes. The genes whose expression is associated with smoking-cessation are enriched in genes that function in xenobiotic metabolism including CYP1A1 and CYP1B1. Activation of the aryl hydrocarbon receptor (AHR) signaling pathway by components of tobacco leads to increase in expression of a number of genes such as CYP1B1 and CYP1A1 which are involved in the metabolism of large number of compounds including cigarette smoke components^10,11^. The genes whose expression is associated with smoking-cessation are also enriched in genes that function in homeostasis including MUC2 and MUC13. These genes encode for members of the mucin protein family and are secreted onto mucosal surfaces in the airways, playing a major role as a barrier to trapping inhaled microbial organism, particulates, and oxidative pollutants. The mRNA expression levels of these genes have not only been previously detected in airways of the lungs^12^ but there is also evidence that these antioxidants serve to protect the airways from the damaging effects of foreign substances^13,14^. In our dataset, MUC2 and MUC13 were among the genes that were up-regulated in response to tobacco smoke exposure and rapidly reversed upon smoking-cessation, causing a decrease in production of mucous in the nasal epithelium. While previous studies indicate that most of these genes are induced in cigarette smoking^3,5,15^ and revert to baseline in long-term smoking-cessation^4,5^, we have found that these genes are amongst the most rapidly reversible following smoking-cessation and that they revert towards baseline levels within 4 weeks following smoking-cessation.

We have shown two different kinetics with which genes are altered in response to smoking-cessation. Most of the genes are altered at 8 weeks of cessation (88.2%) and a subset is altered at 4 weeks of cessation (11.8%). In our dataset, CYP2A6 is another gene among the genes that were downregulated in response to smoking-cessation. CYP2A6 is a human cytochrome P450 2A6 known for metabolizing a number of toxicologically significant chemicals in tobacco smoke including *N*-nitrosonornicotine (NNN), 4-(methylnitrosamino)-1-(3-pyridyl)-1-butanone (NNK) and nicotine^10,11^. 70–80% of nicotine is metabolized by CYP2A6. Interestingly, it is known that there is major variation in CYP2A6 enzyme activity between individuals which effects the rate of metabolism of CYP2A6 substrates. The gene that encodes the CYP2A6 enzyme is highly polymorphic and constitutes more than 40 variants. Moreover, variation in CYP2A6 activity has been shown to be associated with several smoking-related phenotypes. Given the variants, individuals can be genotyped and can be sorted into different groups depending on the activity of CYP2A6 suggesting CYP2A6 expression is an important clinical consideration^16^. It may therefore be interesting in future studies to determine if variability in the kinetics of changes in CYP2A6 expression following cessation are associated with variants that alter CYP2A6 activity. While the majority of nasal epithelial gene-expression levels decrease with smoking-cessation, a small number of genes with important biological implications show increased expression. These include genes involved in repressing apoptosis, such as baculoviral IAP repeat-containing protein 1 coding gene, NAIP, which is involved in inhibiting the activity of apoptotic proteins^17^. Collectively, we have seen that these genes are altered within 8 weeks following smoking-cessation.

Importantly, these smoking-cessation associated genes are among the genes most altered between current and never smokers (in both nasal and bronchial epithelium samples)^5,8^. Moreover, genes that are changed in one direction by smoking are changed in the opposite direction during cessation. We also find concordance between genes differentially expressed upon cessation and gene expression changes associated with *in vitro* short-term exposure to tobacco smoke in airway epithelial cells^5^. Taken together, these data argue that longitudinal gene expression changes in response to short-term recovery from tobacco exposure delineate two different kinetics, majority changing with 8 weeks of cessation and a subset changing with 4 weeks of cessation, and further are concordant with directions of change predicted by *in vitro* and *in vivo* datasets examining gene expression alteration associated with smoke exposure.

One of the strengths of our study is the intra-individual repeated-measures design, which provides additional statistical power compared to cross-sectional observational studies. Nevertheless, our study was limited to 8 smokers who remained tobacco abstinent for the entire 24 weeks period. Future studies profiling a larger number of individuals for a longer period of time after smoking-cessation would help to identify longer-term kinetics of the response to cessation, inter-individual variability in response to tobacco cessation, and disease risk. Furthermore, while this study was designed to examine nasal gene-expression profiles that change during smoking-cessation, it was not designed to address smoking-induced changes in nasal gene expression that are unaltered by cessation. Finally, additional studies are needed to broadly evaluate the possible non-linear changes of nasal gene expression during tobacco recovery, which was challenging to accomplish with the limited samples in this study.

In summary, we have identified longitudinal changes in gene expression from the nasal epithelium of individuals in the process of quitting smoking and identified functional differences in genes with different post-cessation expression kinetics. The majority of the genes we identify as being altered by smoking-cessation change within 8 weeks of cessation while a subset is altered even more rapidly (within 4 weeks). We found that many of the genes that are altered in response to smoking-cessation in the nasal epithelium are the converse of the airway gene expression changes identified between current and never smokers or following short-term exposure of airway epithelial cells to cigarette smoke *in vitro*^18^. This suggests that many of the genes that are altered after 4 to 8 weeks of smoking-cessation are among the genes that are rapidly induced in response to tobacco smoke exposure. Moreover, our data suggest that nasal epithelium might serve as a minimally-invasive tool to measure the reversible impact of smoking in population-based studies.

## Methods

### Patient population

Active smokers enrolled in a smoking-cessation program at Boston University Medical Center (BUMC), Boston MA and a cessation trial at the University of Minnesota (UMN), Minneapolis MN were recruited for this study (Fig. 1). The UMN trial consisted of three arms in which subjects stopped smoking their regular brand of cigarettes at baseline and used one of the following methods for 8 weeks: (1) nicotine patch, (2) an experimental low-nicotine cigarette, or (3) both patch and low-nicotine cigarette. Only subjects in the nicotine patch study arm were included in this analysis. At both BUMC and UMN, brushings were obtained at baseline and at 4, 8, 16, and 24-weeks follow-up. Detailed questionnaires were used to assess tobacco abstinence at each visit. Tobacco abstinence was confirmed either by a urine cotinine level less than 10 ng/mL during treatment for participants not using Nicotine Replacement Therapy (NRT), exhaled carbon monoxide (CO) level less than 10ppm (UMN subjects receiving NRT), or required levels of serum anatabine and anabasine (BUMC subjects receiving NRT). Cotinine, anatabine and anabasine assays were performed at the University of California-San Francisco (UCSF) and University of Minnesota. Subjects in whom abstinence could not be confirmed by the above methods were excluded from the study. The study was approved by the Institutional Review Boards at BUMC and UMN and performed in accordance with all relevant guidelines and regulations. All subjects provided written informed consent.

### Nasal epithelial sample collection

Nasal airway epithelial cells were collected by brushing the inferior turbinate as previously described^8^. Briefly, the right nare was lavaged with 1cc of 1% lidocaine. Using a nasal speculum (Bionox, Toledo, OH) to spread the nare, a standard cytology brush (Cytosoft Brush, Medical Packaging Corporation, Camarillo CA) was inserted underneath the inferior nasal turbinate. Once in place, the brush was rotated once, removed, and immediately placed in 1 mL RNAprotect Cell Reagent (Qiagen). The samples were stored at −80 °C until processing. After thawing the samples, RNA was isolated using the miRNeasy Mini and RNeasy MinElute Cleanup kits as per the manufacturer’s protocol. Integrity of the RNA was confirmed on Agilent 2100 Bioanalyzer using an RNA 6000 Pico Kit. All experimental protocols were approved by BUMC and carried out under relevant guidelines and regulations.

### Genome-wide gene expression profiling

200 ng of high molecular weight RNA was processed and hybridized to Affymetrix Human Gene 1.0 ST Arrays (Affymetrix, Santa Clara, CA). Human Gene 1.0 ST CEL files were normalized to produce gene-level expression values using the implementation of the Robust Multiarray Average (RMA)^19^ in the affy package included in the Bioconductor software suite^20^ and an Entrez Gene-specific probeset mapping (14.1.0) from the Molecular and Behavioral Neuroscience Institute (Brainarray) at the University of Michigan^21^. Probeset summarization, log2 normalization, and generation of relative gene-expression levels were all performed in the R statistical environment (R 2.13.1).

Principal component analysis (PCA) of all genes across all samples were used to assess microarray data quality^19,22^. Samples with 1.5 standard deviation from the mean of each principal component (PC1 and PC2) were identified as low-quality samples and were excluded from the analysis. Baseline samples that appeared as outliers by the above criteria had their subsequent timepoint samples removed.

### Modeling the effect of smoking-cessation

Gene-expression levels associated with tobacco recovery over 24 weeks were identified using analysis of variance (ANOVA) from linear mixed effect regression models^23^. We included a fixed term for log-transformed time in weeks and controlled for RIN (a measure of RNA quality; fixed effect) and inter-patient variability (random effect). A pseudocount of 1 was added to all time points prior to log transformation.

The full model (1) was compared to the reduced model (2) for each gene using ANOVA.

1 $${{\rm{gene}}}_{{\rm{ij}}} \sim {{\rm{\beta }}}_{{\rm{0}}}+{{\rm{\beta }}}_{{\rm{Time}}}\,\ast \,{{\rm{X}}}_{{\rm{Time}}}+{{\rm{\beta }}}_{{\rm{RIN}}}\,\ast \,{{\rm{X}}}_{{\rm{Rin}}}+{{\rm{\varepsilon }}}_{{\rm{ij}}}+{{\rm{\varepsilon }}}_{{\rm{patientj}}}$$

2 $${{\rm{gene}}}_{{\rm{ij}}} \sim {{\rm{\beta }}}_{{\rm{0}}}+{{\rm{\beta }}}_{{\rm{RIN}}}\,\ast \,{{\rm{X}}}_{{\rm{Rin}}}+{{\rm{\varepsilon }}}_{{\rm{ij}}}+{{\rm{\varepsilon }}}_{{\rm{patientj}}}$$

The FDR was calculated for each ANOVA p-values using the method of Benjamini and Hochberg^24,25^. A threshold of ANOVA FDR < 0.05 and a linear fold change (FC) ≥ 1.7 was used as the threshold of statistical significance. In addition to modeling time as a continuous linear variable, gene-expression differences associated with smoking cessation were also identified using an approach where cessation time was modeled as a fixed binary variable representing the switch from baseline to recovered using linear mixed effect regression models while also controlling for RIN (fixed effect) and patient effects (random effects). The specific binary models tested included a switch from baseline to recovered at either 4 weeks, 8 weeks, 16 weeks or 24 weeks (i.e. the samples prior to the critical timepoint are coded as “baseline” and the samples at the critical timepoint and later are coded as “recovered”). As our analyses demonstrated that most genes with altered expression after smoking cessation change their expression at either four- or eight-weeks, we also explored whether there might be transient gene expression differences at the four- or eight-week timepoints using models in which samples at the four- and/or eight-week timepoints are coded as “recovering” and samples at the other timepoints are coded as “baseline”.

Further characterization of the genes whose expression levels were associated with smoking-cessation was performed using hierarchal clustering of all samples supervised by their time points using differentially-expressed genes.

### Experimental validation by quantitative real time PCR

Quantitative RT-PCR analysis was used to validate the expression levels of three genes associated with smoking-cessation: ALDH3A1, CYP1A1 and CYP1B1. The analysis was performed using SYBR Green-based RT² qPCR Primer Assays (Qiagen). GAPDH was used as the housekeeping gene to normalize all samples. Briefly, RNA samples were treated with gDNA Elimination Buffer to remove any contaminating genomic DNA and reverse transcribed with a mix of random hexamers and oligo-dT primer to generate first-strand cDNA, using Qiagen’s RT² First Strand Kit. PCR amplification mixtures (25 μl) contained 9 ng of template cDNA, 12.5 μl of 2 × RT² SYBR Green master mix (Qiagen) and 400 nM RT² qPCR primers. Forty cycles of amplification and data acquisition were carried out in StepOnePlus Real-Time PCR systems (Applied Biosystems). Threshold determinations were automatically performed by StepOne Software (version 2.2.2; Applied Biosystems) for each reaction. All real-time PCR experiments were carried out in triplicate on each sample. Relative gene-expression levels were calculated using the comparative C_T_ method^26^.

### Comparison of smoking-cessation genes to other smoking datasets

GSEA was used to identify the relationships between gene-expression levels associated with duration of smoking-cessation and previously 3 published datasets using the GSEA v2.0 software^9^. Raw data or processed data was obtained from National Center for Biotechnology Information (NCBI), Gene Expression Omnibus (GEO) for the following studies GSE16008^8^, GSE7895^5^, and GSE10700^18^. Significant enrichment was defined by an FDR corrected p-value < 0.05. Further information about these datasets and their processing is provided (see Supplementary note, Connection to other datasets).

### Functional Kinetics of Genes Associated with Smoking-cessation

DAVID (version 6.7) was used to identify Gene Ontology (GO) molecular function categories and KEGG pathways that were over-represented among genes whose expression levels are considerably associated with the duration of smoking-cessation^27^. A p-value ≤ 0.05 was used to determine significant enrichment. For similar functional categories, the union-set of enriched genes was taken from the GO terms and KEGG pathways. Similarity of functional categories was determined based on major overlap of genes (>50% within a category that were also found in another similar category, see Supplementary Table S3) enriched for each functional category.

To determine the gene-expression kinetics for different functional categories of genes, a metagene score was calculated for each enriched functional category. The metagene score was determined by the first principal component of the z-score normalized expression levels of genes associated with smoking-cessation in an enriched functional category. The directionality of functional categories consisting of a combination of up and down-regulated genes was assigned based on literature search of the genes and their general direction of expression in that specific pathway. Differences in metagene scores were assessed using ANOVA and linear mixed effects model adjusting for RNA quality and random patient effect.

### Accession codes

Gene Expression Omnibus (GEO) (GSE83364).

## Supplementary information

Supplementary Info

Supplementary Dataset

## Data Availability

Raw and processed microarray data have been deposited in the Gene Expression Omnibus (GEO) (GSE83364).
