# Supplementary material for: Tobacco-Related Alterations in Airway Gene Expression are Rapidly Reversed Within Weeks Following Smoking-Cessation
Source: Sci Rep. 2019 May 6;9:6978. doi: 10.1038/s41598-019-43295-3 (PMC6502805; doi:10.1038/s41598-019-43295-3)
Supplement: Supplementary file 1 — Supplementary Info [file 41598_2019_43295_MOESM1_ESM.docx]

**Supplementary Material**

**Title:** Tobacco-related alterations in airway gene expression are rapidly reversed within weeks following smoking-cessation

**Author List:** Kahkeshan Hijazi, Bozena Malyszko, Katrina Steiling, Xiaohui Xiao, Gang Liu, Yuriy O Alekseyev, Yves-Martine Dumas, Louise Hertsgaard, Joni Jenson, Dorothy Hatsukami, Daniel R. Brooks, George O’ Connor, Jennifer Beane, Marc E. Lenburg, Avrum Spira

**Supplementary Table S1.** Linear mixed effects model and hierarchal clustering were used to characterize the kinetics of the genes that

revert with short-term smoking cessation. The table shows genes classified into two kinetics following smoking-cessation.

| **Genes reverting in 4 weeks of smoking-cessation** | **Genes reverting in 8 weeks of smoking-cessation** |
| --- | --- |
| ATP6V1C2, AZGP1, BPIL1, CYP1A1, CYP1B1, FAM177B, GLDN, MUC13, MUC2, SMU1, TCP11L2, TFF1, TIPARP,UGT1A6 | AGED2, AGR3, ALDH3A1, ANG, ATP2C2, ATP7B, BNIP3, C14orf45, C1orf110,C3orf25, C9orf116,CA12, CAPN9, CCDC146,CD1A, CES1, CHST9, CLDN22, CLMN, CSPP1, CTGF, CYP2A6,CYSLTR2, DNAJC10, DSG3, FAM126A, GCLC,GNA14, GOLGA8B,GPR115, HBEGF, HEPACAM2,HLA−DQA2, IFT57,IGHV1−18,IGHV3−9, IL1RL1, IQCG, KIAA1147, LDLRAD1, LMAN1, LOC100287841, MAPK10, MAPRE3, MIR27B,NAIP,NME5, NQO1, NUCB2,OLR1,OR14J1,OR4M2, OR52J3,OR5T2, PARVA, PCOTH,PDE4B, PDE8B, PLCB4, PLUNC,PSG1, RAP1GAP, RNASE4, RP1, RPF2,SAA1, SCARNA4, SCARNA9, SCGB2A1, SERPINB2,SERPINB9, SLC35D2, SLC7A2, SNORA1, SNORA20, SNORA2A, SNORA37, SNORA40, SNORA60, SNORA62,SNORD116−1, SNORD116−20, SNORD116−23, SNORD116−24, SNORD116−29,SNORD116−4,SNORD116−6, SNORD116−8, SNORD14E, SNORD38B, SNORD82, SNTN, SPA17, STOX1, TBC1D8, TCTN2, TOX3,TRAV13−2,TRGV5, TSPAN8, VTRNA1−1, ZKSCAN1, ZNF204P, ZNF474, ZNF91 |


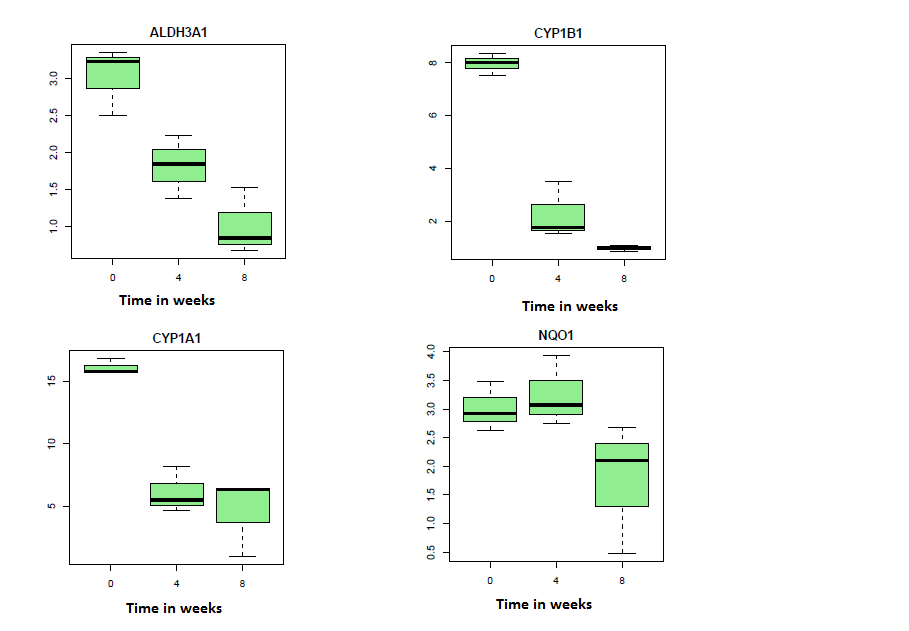


**Log2 relative expression level**

**Log2 relative expression level**

**Log2 relative expression level**

**Supplementary Fig. S1. Quantitative real time PCR results for selected genes across three initial time points from 3 individuals.**

The log2 relative expression level on the y-axis is the ratio of the expression level of a particular sample versus that of a dummy reference sample and on the x-axis is time with baseline samples following 8 weeks of smoking-cessation for 3 individuals (9 samples). The plots are of genes that reverse with rapid kinetics, CYP1B1 and CYP1A1 and ALDH3A1 that reverse with gradual kinetics. These genes were also enriched among an important functional category, metabolism of xenobiotics and anti-apoptosis.

**Supplementary Table S2**. Step model non-linear models.

| **Model Type** | **Time Variable** | **FDR<0.05** | **%age Overlap =**  **Overlapping Genes between A&B**  **-----------------------**  **Total Genes in A** |
| --- | --- | --- | --- |
| **A** | **0481624 (linear)** | **3452** |  |
| **B** | **01111 (non-linear)** | **1** | **1/3452 = 0.029%** |
| **B** | **00111 (non-linear)** | **6373** | **3206/3452 = 92.8%** |
| **B** | **00011 (non-linear)** | **0** | **0** |
| **B** | **00001 (non-linear)** | **0** | **0** |
| **B** | **01000 (non-linear)** | **0** | **0** |
| **B** | **01100 (non-linear)** | **0** | **0** |
| **B** | **00100 (non-linear)** | **0** | **0** |

**Supplementary Table S3**. Functional terms among genes significantly associated with smoking-cessation. (p-value ≤ 0.05).

| **No.** | **Functional Terms** | **Functional Category** | **Categories** | **Genes enriched in functional category** | **Direction of overall gene expression regulation** |
| --- | --- | --- | --- | --- | --- |
| **1** | KEGG Pathways | Metabolism of xenobiotics by cytochrome P450 | Metabolism of xenobiotics by cytochrome P450, Steroid hormone biosynthesis, Retinol metabolism | UGT1A6, CYP1B1, CYP1A1, CYP2A6, ALDH3A1 | down-regulated |
| **2** | GO-molecular terms | Anti-apoptosis | Anti-apoptosis, negative regulation of cell death, apoptosis, negative regulation of programmed cell death | NME5, SERPINB9, GCLC, SERPIN B2, BNIP3, NAIP, MUC2, IFT57, NQO1 | up-regulated |
| **3** | GO-molecular terms | Homeostatic process | Homeostatic process, extracellular region | MUC2, PLUNC, OLR1, RNASE4, IL1RL1, DNAJC10, PSG1, BPIL, AZGP1, SCGB2A1, SAA1, CTGF, ANG, CHST9, UCB2, SERPINB2, HBEGF, TFF1, AGR3, MUC13, GCLC, BNIP3, ATP7B | down-regulated |
| **4** | GO-molecular terms | Response to wounding | Response to wounding, response to inorganic substance | CYP1A1, OLR1, SAA1, CTGF, SERPINB2, HBEGF, LMAN1, GCLC, TFF1, ATP7B | down-regulated |
| **5** | GO-molecular terms | Nucleotide biosynthetic process | purine nucleotide biosynthetic process, purine nucleotide metabolic process | NME5, ATP6V1C2, ATP2C2, PDE8B, ATP7B | down-regulated |
| **6** | GO-molecular terms | Extrinsic to membrane | extrinsic to membrane | GNA14, GOLGA8B, CYP1B1, CYP1A1, RAP1GAP, NUCB2, CYP2A6, SPA17 | down-regulated |
| **7** | GO-molecular terms | Ribonuclease activity | ribonuclease activity | AZGP1, RNASE4, ANG | down-regulated |

**Connection to other datasets**

Details of previously 3 published datasets that were used to identify relationships between gene expression levels associated with duration of smoking-cessation and using the GSEA v2.0 software (Subramanian et al. 2005).

**GSE16008**

Processed gene-expression data was downloaded from GEO for analysis. This data consists of bronchial epithelial cells and nasal epithelial cells collected from healthy current and never smokers. This was a cross-sectional study that was used to identify relationships between smoking-related gene expression changes in bronchial and nasal epithelium within the same individual (Zhang et al. 2009). Genes that are differentially expressed in response to tobacco smoke exposure in both nasal and bronchial epithelial cells, were identified using a mixed linear effect model that included smoking status and site (nasal vs. bronchial) as main effects, and patient and batch as random effects as mentioned previously. For our GSEA analyses, genes were ranked based on the coefficient of the main effect “smoking status,” after eliminating genes significant for the interaction term, representing genes changed by smoking commonly in the bronchus and nose. Enrichment of genes whose nasal epithelial expression levels were significantly associated with the duration of smoking-cessation in the ranked list described above was conducted by GSEA.

**GSE7895**

This dataset consists of gene expression profiles from cytologically normal bronchial epithelium obtained from never, former, and current smokers (Beane et al. 2007). CEL files obtained from GEO were normalized using the RMA algorithm and Entrez Gene CDF v11.0. Two separate linear regression model including terms for age and smoking status were used to identify genes differentially expressed between current and former smokers (the set includes long-term former smokers) and for current and never smokers in the bronchial airway. Two separate rank lists were generated. Genes were ranked according to the strength of their association with 1) current versus former and 2) current vs. never smoking status as determined by the t-statistics from linear model. GSEA was used to compare these ranked lists to the genes whose nasal epithelial expression levels were significantly associated with the duration of smoking-cessation.

**GSE10700**

In this dataset, NHBE cells were treated with whole cigarette smoke from either reference cigarettes (2R4F, University of Kentucky) or a typical American brand of "light" cigarettes ("Lights") for 15 minutes in-vitro and alterations to the transcriptome were assessed at 2, 4, 8 and 24 hours post-exposure using Affymetrix HGU133 Plus 2.0 microarrays (n=52) (Jorgensen et al. 2008). Raw data were normalized using the RMA algorithm and Entrez Gene CDF v11.0.1. Using R, Pearson correlations were computed between gene expression levels for the combined reference and light cigarettes and the time post-exposure. Genes were ranked based on the correlation metric indicating the strength of association of gene-expression changes with time after exposure to whole cigarette smoke. GSEA was used to compare this ranked list to the in vivo smoking cessation signature.

**References**

Beane J, Sebastiani P, Liu G, Brody JS, Lenburg ME, Spira A. 2007. Reversible and permanent effects of tobacco smoke exposure on airway epithelial gene expression. Genome Biol 8:R201; doi:10.1186/gb-2007-8-9-r201.

Jorgensen E, Stinson A, Shan L, Yang J, Gietl D, Albino AP. 2008. Cigarette smoke induces endoplasmic reticulum stress and the unfolded protein response in normal and malignant human lung cells. BMC Cancer 8:229; doi:10.1186/1471-2407-8-229.

Subramanian A, Tamayo P, Mootha VK, Mukherjee S, Ebert BL, Gillette MA, et al. 2005. Gene set enrichment analysis: a knowledge-based approach for interpreting genome-wide expression profiles. Proc Natl Acad Sci USA 102:15545–15550; doi:10.1073/pnas.0506580102.

Zhang X, Sebastiani P, Liu G, Schembri F, Zhang X, Dumas YM, et al. 2010. Similarities and differences between smoking-related gene expression in nasal and bronchial epithelium. Physiol Genomics 41:1–8; doi:10.1152/physiolgenomics.00167.2009.
